# Dr. C. A. Simpson

**Published:** 1872-01

**Authors:** 


Dr. C. A. Simpson.—We are pleased to announce the return of Dr. C. A. Simpson to the city, from New York, where he has been spending some time in perfecting himself in diseases of the eye and ear, to which we learn he intends devoting himself *exclusively* for the future. With his European professional education, and his assiduous and laborious study of the diseases mentioned since his return to the United States, coupled with his high character as a reliable and intelligent gentlemen, we anticipate for him a highly successful future in the interesting and important departments to which he has determined to devote himself.

